# Ten simple rules for writing a peer review

**DOI:** 10.1371/journal.pcbi.1014359

**Published:** 2026-06-15

**Authors:** Farzana Rahman, Dan DeBlasio

**Affiliations:** 1 School of Computer Science and Mathematics, Faculty of Engineering, Computing and the Environment, Kingston University London, London, United Kingdom; 2 Ray and Stephanie Lane Computational Biology Department, School of Computer Science, Carnegie Mellon University, Pittsburgh, Pennsylvania, United States of America; Dassault Systemes BIOVIA Ltd, UNITED STATES OF AMERICA

## Introduction

Peer review is the cornerstone of scientific progress, and it does not exist without the “peers”. Thus, it is part of our responsibility as members of the scientific community to review manuscripts (as well as poster and talk abstracts, funding proposals, theses, white papers, etc.). Doing so can be a daunting task for novice reviewers. While many years are spent increasing our *scientific* expertise, the training that is needed on how exactly to review these important documents is often learned through practice; at times with little guidance. In addition to those new to the process (senior graduate students all the way to junior faculty members) seeking guidance on how to approach the task of peer review; experienced reviewers can always use some “continuing education” in the form of a reminder of what is important when reviewing others’ work.

One of the earliest articles in this series is Phillip E. Bourne and Alon Korngreen’s “Ten Simple Rules for Reviewers” [1]. In it, they provide broad steps on how we, as reviewers, fit into the broader context of the peer-review process. They discuss the implications of choosing *to* review extensively. But one oversight of that article is the actual rules for *constructing* the review, which has nuance. Bourne and Krongreen’s original article didn’t have the room to dig into the nitty-gritty of the actual review, so we hope this list works in concert with that and others in this series.

In this article, we hope to provide an easy-to-read list of rules that we can all reference when reviewing manuscripts. These rules are intended to make sure we’re all living up to the expectations of the peer review process, not only keeping its integrity but also making it a useful exercise both for the original author as well as the reviewers themselves. The ordering of the rules is not in any way a ranking of importance; other than the most important which we leave to last. We tried to organize it in a way that trainees writing reviews can internalize the rules easily and come back to them before, during, and after construction of the actual review. These are the rules as they stand in 2026, but as publishing and peer-review keep changing, these are likely to as well.

### A note about some venue and submission-specific caveats

The‌‌ goal of these rules is to provide a general framework for providing a constructive and useful feedback to the submitting authors. Because of this, some of the details of the specific rules may need to be adapted to the particular venue to which the item was submitted (and in turn the submission type within the venue). In order not to distract from the article overall, we include first the general advice for the current majority of conferences and journals as we see them in 2026. But at the end of some rules we provide a bit of context, as we’re doing here, to point out places where particular venues may have differing advice that reviewers should be on the lookout for.

## Rule 1: Consider your expertise when reviewing

While early review assignments might come from someone very close to you (for example, many times this comes from your advisor in the form of a request to be a sub-reviewer for a conference), there will be some instances where you’re contacted to review by a more distant connection. Before agreeing to review, consider your expertise, time availability, and potential conflicts of interest. Accept only if you can provide a balanced, timely, and informed evaluation. Declining quickly when inappropriate helps editors identify alternative reviewers without delay.

Two points to keep in mind are that: (1) the editors are asking you because they are confident in your abilities to review, while impostor syndrome is real make sure you’re taking this into account; and (2) if you must decline, you have the opportunity to point out others in your subdiscipline who may also be experts but may not be on the editors radar, consider recommended peers who could benefit from the reviewing experience.

When in doubt, don’t be afraid to contact the requesting editor directly. In a diverse discipline such as computational biology, editors may specifically be asking you to review because of your expertise that only applies to a single aspect of a submission. It may not be unreasonable to double-check that, for instance, you’re able to speak to the algorithmic elements of a paper, not the details of the biological procedure introduced to produce the data. An editor can confirm if they, maybe, already have another reviewer who can do the opposite. This is especially common in niche or new problems, where to avoid conflicts of interest all other experts are excluded from review.

It is also important, as mentioned later, to remember that you may also be asked to review a resubmission of this work. The decision becomes easier, but it is not always simple. One thing that might make this easier is a well-structured review (detailed in Rule 7).

Accepting‌‌ the responsibility of review is not a trivial question, and while it is one rule in this list, it has many nuances in and of itself. To that end, there is a whole separate set of rules for this step [1] that goes into much more detail than we do here.

## Rule 2: Respect confidentiality

A submitted manuscript is privileged information. Do not share it, discuss it, or use its data and ideas without explicit permission. Treat the content with the same confidentiality as you would wish for your own unpublished work.

In modern academia, the use of pre-publication release of manuscripts (i.e., arXiv, bioRxiv, etc.) has increased. While this may allow you to share a similar version (don’t share the review copy as some things may be withheld in the preprint) of the contents you are reviewing, your role as a reviewer may not necessarily be public.

At the same time, consider your role/anonymity as the reviewer. In most cases, blind reviewing is an important factor in accepting the review, especially for relatively junior members of the field. Don’t do anything that may compromise this for yourself if it is important to you. On the other hand, some reviewers choose to sign a review, and this is okay too; but in either case it is important to fully understand the implications [2] of the decisions.

At many venues, you may be asked to test software or other tools for replicability. This is not always a trivial task. One common strategy is to make use of virtual machines and dummy accounts. Software and tools should be open and freely available for the most part, so anonymous download is generally preferred if you want to stay anonymous as the reviewer. In some cases, incognito mode (or similar) would be helpful to stay untracked. When testing, if an error message or compile error needs to be included in the review text, it’s best practice to double-check that user names are removed from the error paths, etc. If a login is required to access data that a journal is asking you to review, again you can contact the editor for the specific policies of the venue.

One important aspect of this is the use of LLMs or AI for reviews. This is almost always strictly forbidden, i.e., for this journal the rule is clear [3]. AI agents rarely run locally, and thus by submitting any part of a piece of work to them you’re violating the venue’s privacy policy.

## Rule 3: Be a (reasonably) skeptical scientist

A good review requires more than a single read. First, scan the manuscript for overall structure, contribution, and scope. Then, re-read with focus on methodology, data integrity, and interpretation. A final skim helps ensure coherence in your feedback.

Once again, this rule is complex enough that it has its own article in the “10 simple rules” series [4]. The main difference between reading a manuscript to learn its contents and reading it as a reviewer is a *slightly* higher level of skepticism (more details of this in the remaining rules). While you may read a published paper and be confident that multiple experts have dug into the details, now it’s your turn. If you have a question while reading make sure it gets answered.

As a reviewer, you are the last line of defense in checking the validity of the work. Ask yourself the following as you read in detail (in no particular order):

- Are the claims valid?
- Do the experiments performed answer the initial questions posed?
- Are all figures, tables, and data supported by evidence?
- Are the statistics used valid for the study being performed?
- Are potential confounding factors or sources of bias adequately addressed in the study?
- Is the methodology appropriate and described in sufficient detail to allow replication/reproducibility of the study?
- Are all of the mathematical equations correct, and does the logic flow between them?
- Are the lemmas, theorems, and their proofs correct?
- Have the authors appropriately used FAIR [5] principles?
- Is the study replicable?
- Are there venue-specific questions you need to ask, such as the adherence to open science policies?
- If this is a resubmission, did the authors adequately respond to the previous reviews? (Yours and the other reviewers!)

## Rule 4: Don’t forget the supplemental material

Supplemental material can be as, if not more, important scientifically than the “paper” itself (looking at you Science and Nature). This needs to be reviewed as well for any issues. Ideally, the main paper should be able to be understood without the supplement, part of your task as the reviewer is to ensure this is true. If something is missing or not needed, suggest to the authors that in a resubmission those results are important enough to be moved into the main document or vice versa.

In the context of conference reviewing, they often state that reading the supplement is optional for reviewers, it often contains some of the experiments you may have noted as being missing. Even with that instruction, the material should be skimmed at minimum.

If the reviewers’ instructions note that supplement (or appendix) reviewing is optional, ask why? Are the supplemental materials included in the conference proceedings? Are the supplemental materials going to be trimmed or only available online while the proceedings are on paper? This will help to guide the advice you give to the authors.

## Rule 5: A picture is worth 1,000 words, a figure may be worth more

Examine every figure with a fine-toothed comb. Ensure that all labels and text are clear and legible, and that the data presented are consistent with and accurately support the descriptions in the main text.

Figures are a major component of a manuscript. In some cases, it may be the majority of what a reader, once the manuscript is published, retains and understands. While they can be helpful for exposition, making a good figure is not trivial. Make sure you, as the reviewer, are able to fully internalize all components of the presented figures. This includes the text that accompanies it.

One thing to keep in mind, while we have moved to a mostly digital world, academic publishing makes the humble PDF the ground truth (in most cases). So while you can zoom in a lot on your monitor or tablet to see small details, when printed on a piece of paper that’s not really possible (unless you’re the writers on CSI).

A manuscript’s figures are also a chance to consider all of the readers of a manuscript; are they using screen readers? Are they color blind? Are they not able to print a manuscript in color? Since most of the manuscript is text (and math), all of these are more easily solved, but figures need a little more care. Consider the principles of Universal Design for Learning [6]: designing materials “so they can be used readily by the widest possible range of users”. As an example, just because you make a figure better for someone who is color blind, you make the point more clearer to everyone by making the visual more distinct overall.

## Rule 6: Consider the broader context

Beyond evaluating technical correctness, reflect on the contribution’s significance, reproducibility, and alignment with current literature. Highlight connections to related work and suggest relevant citations when they strengthen, not inflate, the manuscript.

Are there communities of work that the authors may not be aware of that are relevant to this study? This should go beyond just wanting them to cite your papers, but remember making an advancement in science generally requires knowing the state of the art. With our field growing as fast as it is, it’s almost impossible to see everything being published, and while you may be focusing on one domain, you may miss a tangential domain that once distilled down solves the same computational problem. This works both ways: does this work have applications the author is unaware of? Is there a result from another area that can improve this work? If so, point them out in your review.

## Rule 7: Be specific, clear, and well-structured

Vague remarks (“the methods are unclear”) are unhelpful. Instead, identify precise sections, provide examples, and explain why an issue matters (“figure X lacks axis labels, making interpretation difficult”). Evidence-based comments support both authors when updating a manuscript and editors in decision-making.

This also involves making sure your comments are easily understood by the authors. One of the best ways to do this is to make sure the review is well-structured and concise. An *example* of a way to ensure this happens is to use a “summary-major-minor” format for your comments:

- **Summary** — In a short statement at the start of the review, briefly summarize the findings presented in the manuscript as you understand them. This helps the author see how a reader interprets the work (in case it is different from the original intent). Then, in a sentence or two, frame your opinion of the submission, make sure you do not include an indication of your suggested decision. It’s a good idea to include both the good and not-so-good elements.
- **Major Comments** or “must-haves” — Following the summary and high-level opinion, state the major areas of concern. You can think of these as items that are more than just presentation, but something substantial missing or which when altered can make the argument within the paper stronger. It is here where a reviewer would comment on a missing statistic or a small experiment that would confirm an assertion in the manuscript. While not always the case, this is where any “fatal flaws”, which might lead to a rejection or major revision, would be described in detail as an action item the authors can use to update the submission. This list should contain all of the parts of the paper you think need to be added to make it publishable and require another review (by you or another reviewer).
- **Minor Comments** or “nice-to-haves” — Finally, you can include a list of items that should be corrected, but don’t necessarily require extensive effort from the authors, and you would be comfortable with the editor confirming are done without a re-review from yourself. These are things like missing but clear axis labels, formatting of mathematics equations that makes understanding difficult, etc.

Your task at the end of the day is to help the editor decide on what to do and give the authors information on how to improve their work. Be clear about the things that must be done in a resubmission, and those that would be “nice-to-haves”. Most journals will ask reviewers to choose between: accept, minor revision, major revision, and reject (at conferences, you may see the more detailed: strong accept, accept, weak accept, borderline, weak reject, reject, and strong reject). It’s good practice to write the written review before selecting one of these options. If there are only minor comments and some “nice-to-haves”, then it’s probably a minor revisions; any number of “must-have” major comments should trigger major revisions. Make use of the “private comments to the editor” field to be more precise about your expectations if you feel it should be clarified from the author’s feedback. It is highly likely that the editors will ask the same reviewers to re-review major revision submissions.

## Rule 8: Balance criticism with constructiveness

Critical feedback is necessary, but it must be constructive. Point out weaknesses clearly, suggest ways to address them, and acknowledge strengths. A balanced review provides both encouragement and actionable guidance.

One of the best ways to do this is by providing a suggested solution. What can the authors do in a revision to satisfy the issue you have identified? This is a slippery slope, as you don’t want to totally redesign the project, but by providing an example, you have given a direction on something the authors likely overlooked. At the same time, if you suggest a change and the authors decide to satisfy the concern in a different manner, don’t hold that against them in the review of the revision.

Don’t be afraid to point out positives if you see them. If the authors made a major discovery that maybe needs to be emphasized, or the importance is undervalued as written, let them know.

## Rule 9: Keep it relevant, keep it respectful

Reviews should remain professional and respectful. Avoid dismissive or sarcastic language. Be the reviewer you want to get a review from.

Be aware of implicit biases—towards institutions, countries, gender, or research areas—and strive for impartiality. Anonymity should never be used as a shield for discourtesy.

Don’t use the author’s affiliation as a basis for an opinion. Especially true in computational fields like ours, great work does not necessarily need large infrastructural investment. Many modern discoveries are made on a single machine. In some ways its best to not look at the author’s affiliations (or names for that fact) if possible. In some field, review is done double blind, and this solves some things, but when it’s not, do your best to avoid factoring it in.

In the same way, don’t let the author’s background to cloud your judgment of the work. When writing a review, the world outside of science should be ignored. There are no invasions in science; there is no war in science. When there are conflicts in science, it should be about ideas, not political ideation. Leave all these things at the door, and focus on the work at hand.

Just as you should be aware of your implicit (or explicit) biases, you should also be aware of the same in others. After you submit a review, typically you get a copy of the other reviewers’ submissions as well. Don’t hesitate to bring any issues to the attention of the editor as soon as you notice them. This is another use of the “private comments to the editor” field, if you feel another reviewer was being unfair in the first round of comments, and you want to point it out on revision.

## Rule 10: Focus on science, not style

Probably the most important rule on the list, so we left it for last. Your primary responsibility is to assess scientific validity, originality, and impact. While noting major clarity issues is useful, avoid excessive copy-editing. The editor may assign language checks if needed, this can be suggested in the “private comments to the editor” if needed. Focus on whether the science is sound, replicable, and significant.

When giving feedback, avoid statements such as “the authors were wrong when they said X” but instead something like “the manuscript states X, but evidence says Y”. Remember, the authors of the paper you’re reviewing are people too.

In summary, applying these rules will help you develop a disciplined approach to reading and reviewing scientific manuscripts. With experience, this process will become more efficient and more intuitive. An hour spent carefully reviewing a paper can prevent weeks of wasted effort downstream, and this diligence benefits not only the science but also your growth as a reviewer and researcher. As you refine your ability to critically read the literature, also aim to cultivate effective reading and evaluating habits for yourself [7] and to encourage these practices in others. Peer review is not about reviewing the peer; it is about reviewing the work. Good luck, and happy reviewing.
